# Effects of a community pharmacy-based structured medication review on drug-related problems in all-comers with polypharmacy: a randomized, controlled, double-blind, parallel-group trial

**DOI:** 10.3389/fmed.2025.1656595

**Published:** 2025-09-03

**Authors:** Thorsten Bischof, Alexander Schmidt-Ilsinger, Magdalena Hoppel, Anton Kreuzer, Stefanie Briganser, Philipp Saiko, Susanne Ergott-Badawi, Raimund Podroschko, Stephan Moser, Michael Kossmeier, Bernd Jilma, Stefan Deibl, Christian Schoergenhofer

**Affiliations:** ^1^Department of Clinical Pharmacology, Medical University of Vienna, Vienna, Austria; ^2^Austrian Chamber of Pharmacists, Vienna, Austria; ^3^Humanitas Apotheke, Vienna, Austria; ^4^Marco Polo Apotheke, Vienna, Austria; ^5^Austrian Federation of Social Insurances, Vienna, Austria

**Keywords:** medication review, drug-related problems, polypharmacy, pharmaceutical care, healthcare

## Abstract

**Background:**

Medication reviews may help to reduce the burdens of polypharmacy. A medication review type 2a is a structured evaluation of a patient’s pharmacotherapy based on medication history and patient information in a face-to-face interview.

**Methods:**

This multi-center, randomized, patient- and assessor-blind, parallel-group trial was conducted in 14 community pharmacies in Vienna, Austria. Adult outpatients taking ≥8 drugs were eligible. The intervention was a medication review type 2a. At baseline, pharmacists assessed drug-related problems (DRP), a parameter summarizing problematic aspects of pharmacotherapy, in all participants. Pharmacists randomized patients (1, 1) to the intervention, in which they addressed DRPs, or to the control group, in which DRPs were not addressed. A blind pharmacist reassessed DRPs at the second visit after 3–4 months. The primary endpoint was the difference in the number of DRPs between groups after 3–4 months. Secondary endpoints included changes in therapy adherence, health literacy, and number of active ingredients per patient.

**Results:**

Between August 2022 and August 2023, 220 patients (intervention *n* = 110, control *n* = 110) were randomized; 198 completed the primary analysis (intervention *n* = 98, control *n* = 100). A medication review reduced DRPs by ~70% (effect size 0.30, 95% CI: 0.27–0.34) compared to the control group. Therapy adherence- and health literacy-related DRPs decreased significantly by ~60% and ~64%, respectively. Furthermore, a medication review decreased the mean number of active ingredients by ~9% in the intervention group compared to the control group.

**Conclusion:**

A medication review type 2a effectively reduced the number of DRPs.

**Trial registration:**

ISRCTN14052916.

## Background

Polypharmacy has emerged as one of the most pressing topics in modern healthcare systems (1). Continuous advancements in the social determinants of health have contributed to a steady rise in life expectancy (2). The concomitant increase in chronic diseases and multimorbidity has driven an increase in polypharmacy, collectively putting economic pressure on healthcare systems (3).

Although there is no generally accepted definition, polypharmacy is commonly defined as the intake of five or more systemically available drugs (1). This definition has been criticized because optimal treatment of patients frequently requires the intake of numerous drugs. Thus, more recent definitions differentiate between inappropriate and appropriate polypharmacy. The latter is defined as the judicious prescribing of evidence-based medications, limited to those essential for achieving therapeutic goals, while ensuring patient safety (4).

At the patient level, complex pharmacotherapy regimens pose major challenges, especially for the elderly, and are associated with reduced quality of life, poorer adherence, reduced therapeutic effectiveness, potential drug–drug interactions (pDDI) and adverse drug reactions (ADRs) (5, 6). Notably, the number of drugs taken by a patient is the most relevant risk factor for these issues (6).

The implications for healthcare systems are significant. A systematic review including over 100.000 hospitalized patients, estimated that ~5% of all hospitalizations are related to ADRs, with higher rates among older patients (7). The actual burden is probably underestimated, because ADRs are frequently underreported (8). These issues also translate into increased costs for healthcare systems. In 2012, the US Institute for Healthcare Informatics estimated that inappropriate polypharmacy contributes to ~4% of the avoidable healthcare costs, amounting to ~18 billion US dollars worldwide (9).

The implementation of medication reviews in community pharmacies may contribute to mitigate these burdens. Notably, medication reviews are not currently offered routinely by the Austrian healthcare service. According to the Pharmaceutical Care Network Europe (PCNE) definition, medication reviews are structured patient interviews with systematic evaluations of their medication history with the aim of optimizing their drug use by identifying drug-related problems (DRPs) and recommending interventions to improve health outcomes (10). Studies have investigated the effects of medication reviews in different settings and populations, generally demonstrating benefits for patient outcomes, quality of life, disease biomarkers, and healthcare costs (11–13).

However, these studies had similar limitations with regards to their open-label design, lack of adequate controls, and partly a lack of randomization on patient-level, which may limit the robustness of the results. Additionally, the inclusion of homogenous populations may limit the generalizability of the results. These limitations motivated the present study – the first randomized-controlled, patient- and assessor-blind trial to investigate the effects on DRPs of a medication review type 2a in a heterogenous population with polypharmacy.

## Methods

This was a multi-center, randomized-controlled, patient- and assessor-blind, parallel-group trial that took place in 14 community pharmacies in Vienna, Austria. The design, conduct, and reporting of the study adhere to the Consolidation Standards of Reporting Trials (CONSORT) guidelines. The independent Ethics Committee at the Medical University of Vienna (registration number: 2029/2021) approved the study, which complied with the Declaration of Helsinki and the International Conference on Harmonization–Good Clinical Practice guideline. All patients provided written informed consent before enrolment.

### Study population

Eligible patients were male and female patients aged ≥18 years with intake of at least eight systemically available active ingredients. Patients, who previously participated in a medication review were not eligible. The full list of inclusion and exclusion criteria is presented in the Supplementary Table S1.

### Study intervention

According to the PCNE classification, a medication review type 2a is a structured evaluation of a patient’s medicines with the aim of optimizing pharmacotherapy and health outcomes (10). The type of review is a patient-centered analysis that focuses on the prescribed medication, whereas clinical data such as medical history, indications for pharmacotherapy or laboratory data are not considered. The patient interview is the core of the analysis. It typically consists of two parts: (i) an assessment of DRPs; and (ii) personalized recommendations to improve these DRPs in oral and written form (10).

Drug-related problems as defined by the PCNE include dosage errors, duplicate prescriptions, issues with therapy adherence, lack of effectiveness, pDDIs, side effects, improper storage conditions, as well as patient-reported beliefs about their medication and health (14). Therapy adherence-related DRPs were assessed based on self-reported difficulties with remembering or correctly using medications, including questions on the frequency of missed doses, risk of confusing medicines, and irregular usage patterns. Health literacy-related DRPs were identified by assessing whether patients were aware of the indication for each medication and whether they could correctly state their dosing regimen. The complete structure of the medication review type 2a including a detailed breakdown of how specific responses contributed to DRPs is presented in the Supplementary Table S2. Of note, treating physicians were not systematically involved in the study, and in Austria, only physicians are authorized to prescribe, change, or describe medicines in the outpatient setting. However, to adapt pharmacotherapy, pharmacists recommended patients to discuss the result of the medication review with their treating physicians.

A specific software was developed to guide medication reviews within the study. This software covered all aspects of the medication review and additionally served as quality control. The software also featured a pDDI database^1^ that automatically provided pharmacists with a list of pDDIs including a severity grading (1 = contraindicated, 2 = severe, 3 = moderate, 4 = minor) of each pDDI. Less severe pDDIs (5 = product-specific warnings, 6 = no interaction expected, 7 = no statement possible) were not included in the analysis. Furthermore, pharmacists could rule out clinical relevance of pDDIs based on available medical records (e.g., laboratory parameters showing that there is no hyperkalaemia or records on blood pressure). Of note, during the medication review pharmacists may have recommended to perform specific investigations or examinations to assess clinical significance of pDDIs. In addition, pharmacists entered all information into the software, which also served as an electronic case report form that was exported centrally by the investigators at the end of the study. An automated algorithm calculated the identified factors into a composite summative parameter of DRPs.

A total of 28 pharmacists, two from each of the 14 participating pharmacies, completed structured training sessions for this software. These sessions included lectures, case studies, and individual feedback rounds to ensure consistency of the reviews.

### Study procedures

Community pharmacists recruited patients by asking all-comers with intake of at least eight active ingredients to participate in this study. They checked eligibility criteria and obtained informed consent from each patient.

The study essentially comprised two parts: Part 1 investigated the effects of a single medication review in a double-blind manner. Patients completed a baseline visit with an initial assessment of DRPs. Thereafter, pharmacists randomized patients into an intervention arm, in which they performed the second part of the medication review including individual recommendations and explanations to improve drug therapy, or into a control arm, in which the interview ended without providing such recommendations. Pharmacists were specifically trained not to unblind patients at this stage: they arranged an appointment for the next meeting and communicated to the patients that the obtained information would be analyzed in detail until the next meeting. In that context, pharmacists were allowed to answer any patient questions and were required to provide recommendations in cases of dangerous drug combinations or overdoses. The second interview took place 3–4 months after the first interview. An independent assessor – a pharmacist trained in performing medication reviews, who was unaware of the patients’ group allocation – assessed DRPs. Thus, the first and second assessment of DRPs took place in a double-blind manner: the first before randomization, the second with a masked assessor and blind patients. The assessment of the primary endpoint ended at this stage of the trial (Part 1).

In Part 2 of the study, pharmacists offered a medication review to all patients: the second medication review for patients in the intervention group and the first medication review for patients in the control group. All patients were specifically asked whether they wanted to continue or terminate their participation at this stage. Termination of participation after the first part was not treated as a premature termination. This design was chosen to assess the acceptance of the intervention. Pharmacists conducted the final assessment of DRPs 6–9 months after the baseline visit in an open label manner.

In case of missed appointments, pharmacists made at least three attempts to contact patients by phone or e-mail before classifying them as “lost to follow-up.” The end of the trial was defined as the date of the last visit of the last patient participating in this study.

### Outcomes

The primary endpoint was the difference between the intervention and the control group in the number of DRPs at month three-to-four to investigate the effect of a single medication review. Secondary endpoints included (i) the difference in the number of DRPs at month six-to-nine (effect of two medication reviews vs. one medication review), (ii) the effect of medication reviews on health literacy and therapy adherence related DRPs at month three-to-four and six-to-nine, (iii) effects of medication reviews on the number of drugs and active ingredients taken by each patient at month three-to-four and six-to-nine, (iv) correlations between DRPs and demographics or specific baseline parameters. Within the medication review patients were asked about their general health status and whether it had changed recently. These patient-reported outcomes were analyzed exploratory post-hoc. The full list of endpoints is presented in the Supplementary Table S3.

### Sample size

Based on other studies in various populations (15–17), we assumed a mean ± standard deviation (SD) of 3.5 ± 2.1 DRPs per patient in our study and aimed for a 25% reduction, which the investigators deemed clinically meaningful and realistic (16, 18). A sample size of 92 patients per group would have sufficed to show a statistically significant difference with a two-sided alpha error of 5%, a power of 80%, using a two-sample *t*-test (G.Power 3.1). This number was increased to 100 patients per group to account for potential dropouts. Furthermore, the sample size could be increased by 10%, if it was foreseeable that dropout rate exceeded 10% before the second status assessment. The sample size calculation was deemed conservative given that (i) the strict inclusion criterion “intake of at least eight active ingredients” was likely to result in a higher number of DRPs in our population, (ii) larger effect sizes were observed in other studies and (iii) the use of a more efficient statistical test for the primary analysis.

### Randomisation

Pharmacists randomized patients using a permuted block randomization with a size of eight with an interactive web-response system available at www.meduniwien.ac.at/randomizer. Patients were stratified by the number of active ingredients (<10, ≥10). This stratification variable was used due to the demonstrated correlation between the number of drugs taken per person and the number of DRPs (19).

### Statistics

Patient demographics and baseline data were summarized by descriptive statistics (e.g., mean, SD, number (n), and percentage (%), as applicable). Absolute and relative numbers of DRPs, contributors to DRPs, and the impact of medication reviews on various components are presented descriptively. The empirical distribution of DRPs was visually examined using boxplots. Between group differences regarding the primary and secondary endpoints with metric variables were analyzed by a count data (mixed effect) regression model with treatment group and sex as fixed factors, age in years, baseline number of active ingredients, and baseline values of the respective outcome variable as covariates, and each pharmacy as random intercept. Between group differences regarding the number of active ingredients per patient (secondary endpoint) were analyzed as above, but without baseline number of active ingredients as additional covariate. Due to potential overdispersion within our data, negative binomial regression was deemed appropriate and was used for all analyses of primary and secondary endpoints instead of the initially considered Poisson regression. Exploratory endpoints focusing on patient-reported outcomes were dichotomized in a way that one represents the favorable outcome (e.g., “1” for very good/good, “0” for moderate/poor/very poor state of health) and analyzed using logistic (mixed effect) regression models with the same fixed factors, covariates and random intercepts as for the primary analysis. Furthermore, we investigated possible correlations between sex, age and number of active ingredients at baseline with the number of DRPs at baseline using the Spearman rank correlation. Pairwise comparisons of DRPs within each group at the different time-points were calculated by a non-parametric Wilcoxon signed-rank test. For all inferential tests, a two-sided significance level of 0.05 was used. Inferential tests of secondary and exploratory endpoints were not adjusted for multiplicity.

All statistical analyses were calculated with R Version 4.4.1. Missing data were not imputed.

## Results

**Figure 1:**
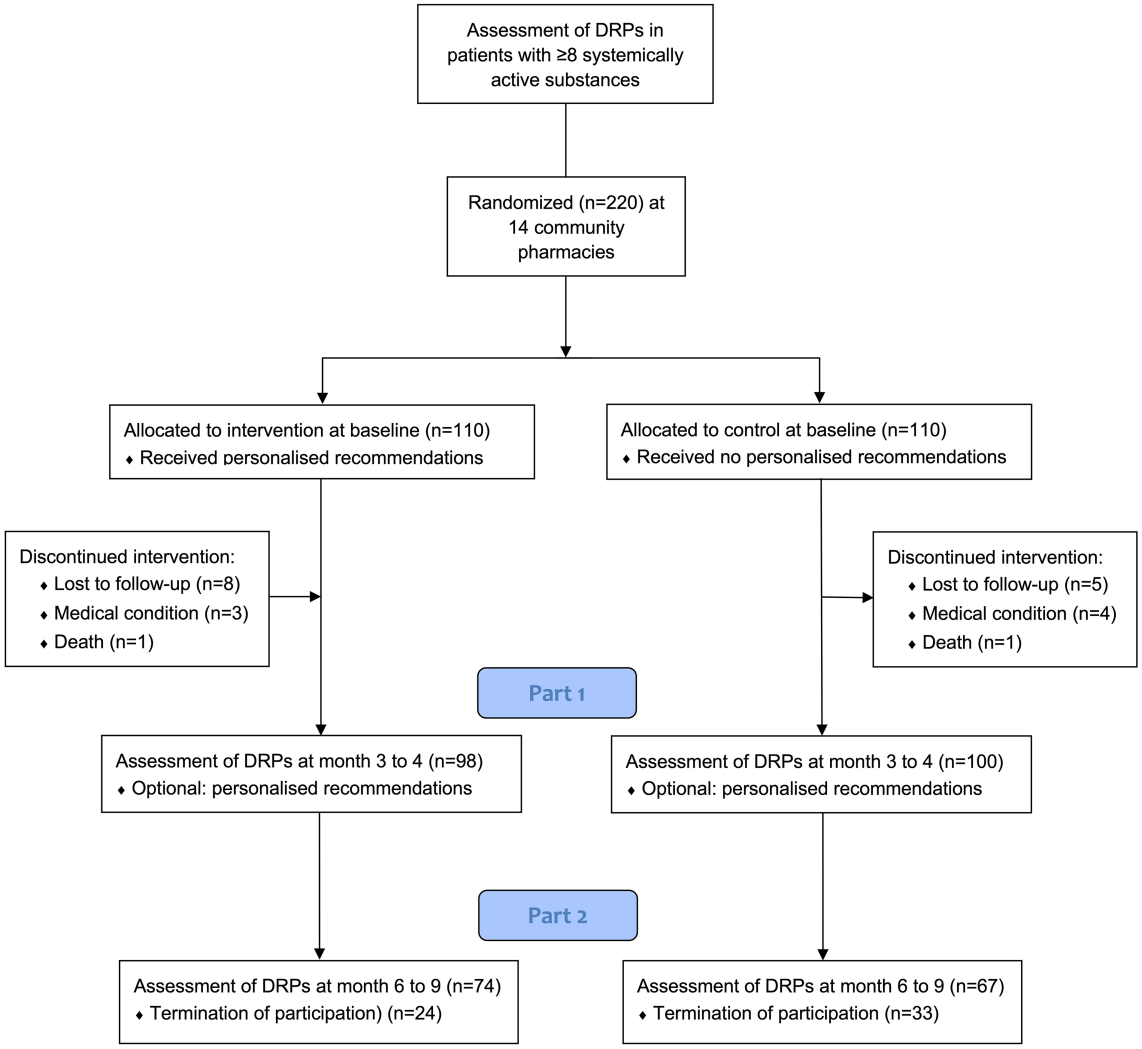
Patients flow diagram. DRPs = drug-related problems. *After completion of Part 1, participants were asked if they wanted to continue the study or if they preferred to terminate it.

Between August 2022 and August 2023, a total of 220 patients underwent randomization; 110 were assigned to the intervention group and 110 to the control group. Of these, 198 (90%) completed Part 1 of the study, with 98 patients assigned to the intervention group and 100 patients assigned to the control group. Twenty-two subjects discontinued the study during Part 1 (Figure 1). A total of 141 (71%) patients also participated in Part 2 of the study, 74 patients in the intervention group and 67 patients in the control group. The last visit of the last patient took place in May 2024. A flowchart of the trial is presented in Figure 1.

Table 1 displays baseline characteristics and demographics of patients, who completed Part 1. For those who completed Part 1 and 2, these data are presented in the Supplementary Table S4. Overall, the groups were well-balanced, however, patients in the control group were on average 3.9 years older than those in the intervention group (*p* = 0.04). Drug-related problems, the primary endpoint of the study, as well as the number of active ingredients taken by each patient, as well as the patient-reported health status, were comparable between both groups. At baseline, the number of active ingredients correlated with the number of DRPs (Spearman correlation, rho = 0.59, *p* < 0.001). However, other baseline parameters, such as age or sex did not correlate with DRPs. Table 2 presents the contribution of individual components to the summative parameter DRPs.

**Table 1:** Demographics and baseline data for patients of Part 1.

Parameter	Units	All patients (*N* = 198)	Intervention (*N* = 98)	Control (*N* = 100)
Age	Mean ± SD	70.6 ± 13.6	68.6 ± 13.5	72.5 ± 13.5
Sex (male)	N (%)	75 (38)	38 (39)	37 (37)
Weight [kg]	Mean ± SD	81.7 ± 18.4	81.1 ± 18.6	82.3 ± 18.4
Smoker	N (%)	40 (20)	22 (22)	18 (18)
Pregnant	N (%)	1 (0)	0 (0)	1 (1)
Medications	Mean ± SD	12.9 ± 3.8	13.2 ± 4.2	12.7 ± 3.4
≥10 medications	N (%)	139 (70)	70 (71)	69 (69)
≤10 medications	N (%)	59 (30)	28 (29)	31 (31)
Active ingredients	Mean ± SD	14.7 ± 4.3	15.0 ± 4.8	14.4 ± 3.8
≥10 active ingredients	N (%)	165 (83)	83 (85)	82 (82)
≤10 active ingredients	N (%)	33 (17)	15 (15)	18 (18)
Baseline DRPs	Mean ± SD	15.7 ± 9.1	15.7 ± 10.0	15.8 ± 8.2
Subjective health situation				
Very good	%	3	4	1
Good	%	25	24	26
Moderate	%	49	46	52
Bad	%	20	20	19
Very bad	%	4	6	2
Subjective health situation changed				
Improved	%	14	16	12
Constant	%	46	42	49
Deteriorated	%	40	42	39

DRP = drug-related problems, SD = standard deviation.

**Table 2:** Mean drug-related problems for each component at each study visit.

Therapy adherence	Baseline	Month 3–4	Month 6–9
Intervention group	0.82 ± 0.92 (5.2)	0.32 ± 0.53 (4.8)	0.34 ± 0.56 (5.1)
Control group	0.69 ± 0.90 (4.4)	0.73 ± 0.86 (4.8)	0.19 ± 0.40 (5.1)

**Health literacy**	Baseline	Month 3–4	Month 6–9
Intervention group	1.42 ± 3.23 (9.0)	0.54 ± 1.27 (8.2)	0.08 ± 0.32 (4.3)
Control group	1.12 ± 2.30 (7.1)	1.26 ± 2.15 (8.2)	0.16 ± 0.48 (4.3)

**Patient-reported effectiveness of medication**	Baseline	Month 3–4	Month 6–9
Intervention group	0.47 ± 0.50 (3.0)	0.22 ± 0.42 (2.2)	0.09 ± 0.30 (1.2)
Control group	0.54 ± 0.50 (3.4)	0.34 ± 0.48 (2.2)	0.04 ± 0.21 (1.2)

**Patient-reported tolerability of medication**	Baseline	Month 3–4	Month 6–9
Intervention group	0.26 ± 0.44 (1.6)	0.08 ± 0.28 (0.9)	0.04 ± 0.21 (1.2)
Control group	0.28 ± 0.45 (1.8)	0.14 ± 0.35 (0.9)	0.04 ± 0.20 (1.2)

**Improper storage condition**	Baseline	Month 3–4	Month 6–9
Intervention group	0.11 ± 0.32 (0.7)	0.02 ± 0.14 (0.1)	0.03 ± 0.16 (0.8)
Control group	0.10 ± 0.30 (0.6)	0.15 ± 0.36 (0.1)	0.03 ± 0.17 (0.8)

**Inappropriate therapy duration**	Baseline	Month 3–4	Month 6–9
Intervention group	0.07 ± 0.30 (0.5)	0.07 ± 0.30 (2.1)	0.04 ± 0.26 (1.2)
Control group	0.06 ± 0.31 (0.4)	0.32 ± 0.71 (2.1)	0.04 ± 0.37 (1.2)

**Dosage error – deviates from prescription**	Baseline	Month 3–4	Month 6–9
Intervention group	0.75 ± 1.30 (4.7)	0.09 ± 0.32 (2.9)	0.11 ± 0.56 (5.1)
Control group	0.95 ± 1.49 (6.0)	0.44 ± 0.82 (2.9)	0.19 ± 0.50 (5.1)

**Dosage error – deviates from SmPC**	Baseline	Month 3–4	Month 6–9
Intervention group	0.86 ± 1.43 (5.5)	0.44 ± 0.73 (8.0)	0.30 ± 0.57 (8.2)
Control group	0.82 ± 1.25 (5.2)	1.23 ± 1.20 (8.0)	0.31 ± 0.61 (8.2)

**Problems with the use/application of medication**	Baseline	Month 3–4	Month 6–9
Intervention group	0.95 ± 1.63 (6.1)	0.14 ± 0.38 (4.9)	0.08 ± 0.36 (1.9)
Control group	0.58 ± 1.14 (3.7)	0.76 ± 1.09 (4.9)	0.07 ± 0.50 (1.9)

**Inappropriate pharmaceutical form**	Baseline	Month 3–4	Month 6–9
Intervention group	0.09 ± 0.46 (0.6)	0.02 ± 0.14 (0.5)	0.0 ± 0.0 (0.0)
Control group	0.05 ± 0.22 (0.3)	0.08 ± 0.27 (0.5)	0.0 ± 0.0 (0.0)

**(Lack of) effect**	Baseline	Month 3–4	Month 6–9
Intervention group	0.67 ± 1.12 (4.3)	0.18 ± 0.51 (4.6)	0.04 ± 0.20 (4.3)
Control group	0.58 ± 1.00 (3.7)	0.70 ± 1.00 (4.6)	0.18 ± 0.55 (4.3)

**Tolerability**	Baseline	Month 3–4	Month 6–9
Intervention group	0.90 ± 1.38 (5.7)	0.25 ± 0.54 (5.4)	0.08 ± 0.32 (5.4)
Control group	1.14 ± 1.39 (7.2)	0.83 ± 1.07 (5.4)	0.21 ± 0.48 (5.4)

**Other problems**	Baseline	Month 3–4	Month 6–9
Intervention group	0.17 ± 0.50 (1.1)	0.02 ± 0.14 (0.5)	0.01 ± 0.12 (1.6)
Control group	0.14 ± 0.43 (0.9)	0.08 ± 0.34 (0.5)	0.06 ± 0.30 (1.6)

**Contraindicated medication**	Baseline	Month 3–4	Month 6–9
Intervention group	0.09 ± 0.35 (0.6)	0.03 ± 0.17 (0.3)	0.0 ± 0.0 (0.0)
Control group	0.17 ± 0.73 (1.1)	0.04 ± 0.20 (0.3)	0.0 ± 0.0 (0.0)

**Duplicate prescriptions**	Baseline	Month 3–4	Month 6–9
Intervention group	0.90 ± 1.02 (5.7)	0.40 ± 0.65 (3.5)	0.28 ± 0.54 (10.5)
Control group	0.63 ± 0.85 (4.0)	0.53 ± 0.76 (3.5)	0.40 ± 0.65 (10.5)

**Clinically relevant potential drug–drug-interactions**	Baseline	Month 3–4	Month 6–9
Intervention group	7.16 ± 5.69 (45.7)	2.00 ± 2.55 (50.3)	1.47 ± 1.84 (49.0)
Control group	7.95 ± 4.96 (50.3)	7.73 ± 5.40 (50.3)	1.88 ± 3.29 (49.0)

Numbers are presented as means ± standard deviations (percentage of total number of drug-related problems). SmPC = summary of product characteristics.

In the primary statistical analysis, the intervention group had significantly fewer DRPs than the control group after 3–4 months, with an effect size of 0.30 (95% confidence interval (CI): 0.27–0.34, *p* < 0.001), corresponding to a ~ 70% reduction in DRPs. Detailed model results of all analyses can be found in the Supplementary Tables S5–S17.

Clinically relevant pDDIs were the main contributor to DRPs accounting for ~50% of all DRPs at each visit (Table 2). To exclude the possibility that the results of the primary analysis were mainly driven by a reduction in pDDIs, we performed a sensitivity analysis excluding this component and the results remained stable (effect size 0.34, 95% CI: 0.29–0.39, *p* < 0.001).

A single medication review significantly improved the secondary endpoints therapy adherence and health literacy. After 3–4 months, therapy adherence related DRPs were significantly lower in the intervention group compared to the control group (effect size 0.40, 95% CI: 0.26–0.61, *p* < 0.001). Likewise, health literacy related DRPs were significantly lower in the intervention group compared to the control group at the second visit (effect size 0.36, 95% CI: 0.22–0.59, *p* < 0.001). After a single medication review, the mean number of active ingredients taken per patient decreased significantly by 9% at the second visit compared to the control group (effect size 0.91, 95% CI: 0.84–0.98, *p* = 0.01).

Furthermore, we investigated the effects of two vs. one medication review on these endpoints at the third visit after 6–9 months. Numerically, DRPs were 20% lower in the intervention group. However, this difference failed to reach a statistical significance (effect size 0.80, 95% CI: 0.61–1.04, *p* = 0.10). Whereas the second medication review did not significantly yield additional benefits for therapy adherence (effect size 1.51, 95% CI: 0.76–2.98, *p* = 0.24) and the number of prescribed active ingredients per patient (effect size 0.96, 95% CI: 0.87–1.06, *p* = 0.42), it further improved health literacy and related DRPs were significantly lower in the intervention compared to the control group (effect size: 0.31, 95% CI: 0.10–0.93, *p* = 0.04).

**Figure 2:**
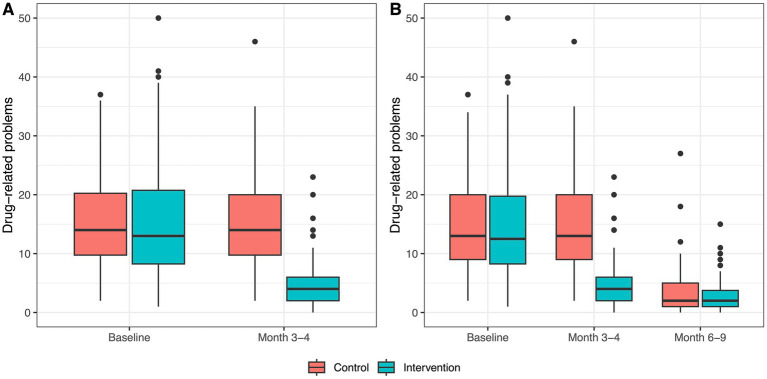
Boxplots of drug-related problems within groups at each study visit. **(A)** Distribution of drug-related problems at baseline and at month 3–4 per group for all patients with data for both study visits (*n* = 198). **(B)** Distribution of drug-related problems at baseline, at month 3–4, and month 6–9 for all patients with data for all three study visits (*n* = 141).

An integral part of each medication review were patient-reported assessments of their personal health status. Compared to the control group, patients in the intervention group were twice as likely to rate their health as “good” or “very good” at the second visit (odds ratio: 2.20, 95% CI: 1.08–4.48, *p* = 0.03). Similarly, when asked about recent changes in their health status, patients in the intervention group were three-times more likely to report improvements (odds ratio: 3.09, 95% CI: 1.40–6.81, *p* = 0.01) (Table 3 and Figure 2).

**Table 3:** Detailed results of a single medication review compared to no intervention after 3–4 months.

Outcomes	Intervention group	Control group	Effect sizes (95% CI)
Drug-related problems			
Baseline	15.7 ± 10.0	15.8 ± 8.2	0.30 (0.27–0.34)
Month 3–4	4.8 ± 4.1	15.4 ± 7.8	
Number of medications per patient			
Baseline	13.2 ± 4.2	12.7 ± 3.4	0.90 (0.83–0.98)
Month 3–4	11.6 ± 4.3	12.4 ± 3.4	
Number of active ingredients per patient			
Baseline	15.0 ± 4.8	14.4 ± 3.8	0.91 (0.84–0.98)
Month 3–4	13.2 ± 4.9	14.1 ± 3.8	
Therapy adherence related drug-related problems			
Baseline	0.82 ± 0.92	0.69 ± 0.90	0.40 (0.26–0.61)
Month 3–4	0.32 ± 0.53	0.73 ± 0.86	
Health literacy related drug-related problems			
Baseline	1.42 ± 3.23	1.12 ± 2.30	0.36 (0.22–0.59)
Month 3–4	0.54 ± 1.27	1.26 ± 2.15	
Patient-reported health – good or very good (%)			
Baseline	27.6	27.0	2.20 (1.08–4.48)
Month 3–4	42.9	30.0	
Patient-reported recent changes in health – improved (%)			
Baseline	16.3	12.0	3.09 (1.40–6.80)
Month 3–4	29.6	12.0	

The numbers in the columns “intervention group” and “control group” are means ± standard deviations (DRPs, number of medications) or proportions (patient-reported outcomes). Effect sizes are either ratios of expected number of DRPs with 95% confidence intervals (CI) from negative binomial (mixed effects) regression models (drug-related problems, number of medications per patient, number of active ingredients per patient, therapy adherence related drug-related problems, health literacy related drug-related problems) or odds ratios with 95% confidence intervals (CI) from logistic (mixed effects) regression models (patient-reported health, recent changes in health).

## Discussion

This study was the first to investigate the effects of a medication review type 2a in a randomized-controlled, double-blind manner. A single medication review significantly reduced DRPs by ~70% compared to the control group. Furthermore, it significantly improved therapy adherence, health literacy, and reduced the number of active ingredients taken by each patient by ~9% compared to the control group. These results corroborate the findings of other studies using similar interventions in unblinded and partly non-randomized studies (15, 16, 20, 21).

A specific strength of this trial was that even patients in the control group had an actual face-to-face appointment with a pharmacist, which stands in contrast to other studies in the field. Although no recommendations were provided, patients answered several questions about their pharmacotherapy. This conversation, but also participation in the study itself, could have prompted behavioral changes, such as improved adherence or increased motivation to learn about their pharmacotherapy. For instance, patients may have scheduled appointments with their treating physicians or obtained information from other sources. Thus, the control group arguably received a “placebo-like” treatment. However, the number of DRPs between the first and second visit in the control group did not change significantly, arguing against a strong placebo effect.

The conduct of the study in a real-world setting in 14 community pharmacies across Vienna with an unselected, heterogenous cohort of all-comers with polypharmacy aimed to achieve high external validity. The burden of polypharmacy was quite high with an average of 13 drugs per patient. This is important because the number of drugs correlates with the number of DRPs, which was also higher in our population compared to other trials (15–18, 20–24). In contrast to other studies, we did not exclusively target elderly patients, but our cohort was 5–10 years younger on average (15–18, 20–24). Notably, routinely performed medication reviews for outpatients are not offered in the Austrian health service. Patients who had previously received a medication review were excluded to maintain blinding of the patients and to include a “treatment-naïve” cohort. However, patients with pronounced polypharmacy and numerous DRPs may have been more willing to participate in this study. Likewise, pharmacists may have preferentially recruited this kind of patients. Furthermore, those with a stronger interest in their personal health may be more likely to participate. Thus, certain degrees of selection and participation bias cannot be ruled out.

The observed reduction in DRPs was more pronounced than in previous studies conducted in similar settings (15–18, 20, 21). This may be attributed to the higher number of DRPs at baseline. Possible explanations include the marked polypharmacy and a comparatively higher number of pDDIs in our study. Across different studies, there are inconsistent definitions about the clinical relevance of a pDDI and what qualifies a pDDI as a DRP (16, 21, 25). Moreover, pDDI databases vary significantly in their severity ratings, and there is a considerable inter-rater variability in assessing clinical relevance of a pDDI (26). In this study, we used a comprehensive approach: pharmacists included all pDDIs identified as clinically relevant by the pDDI database, unless they could be objectively denied based on the respective medical records. This approach aimed to reduce the inter-rater variability and unify the interpretation of pDDIs among the different study sites. However, because pDDIs contributed most points to the primary endpoint, we conducted a sensitivity analysis to investigate the robustness of the findings. Repeating the primary analysis without the component pDDIs yielded a comparable effect size of 0.34 (95% CI: 0.29–0.39), confirming the results. Notably, the estimated effect of the first medication review in the control group was comparable to that of the first in the intervention group, further supporting the robustness.

Educational interventions, such as a medication reviews, offer a valuable role to improve health literacy by increasing patients’ understanding of their disease, as well as the risks and benefits of their treatment, thereby improving therapy adherence (27). In our study, a single medication review reduced the DRPs related to therapy adherence by ~60% and those related to health literacy by ~64%. Systematic reviews have already shown that services offered by community pharmacies may improve such patient competencies (11, 27). Improvements in patient-reported effectiveness and tolerability of medicines may also partly reflect this improved knowledge. Furthermore, interdisciplinary collaboration through pharmacist involvement in patient care has been shown to improve disease biomarkers, such as blood pressure, blood sugar control and cholesterol (28, 29). Although biological parameters as endpoints could not be included in this study, these findings may provide evidence that optimizing pharmacotherapy and reducing DRPs is clinically and practically relevant. The success of the intervention may also be further supported by the results of patient-reported outcomes, first and foremost self-reported health status, which improved after a single medication review. This result may cautiously be interpreted as an improved quality of life.

Another positive result was the ~9% decrease of active ingredients taken per patient compared to the control group. A previous multi-center trial from community pharmacies in the Netherlands reported a ~ 5% reduction per patient, although patients only took ~9 drugs at baseline, suggesting that the observed reduction may be proportional to the initial number of drugs (18). This should simplify the prescription complexity and likely reduce costs. However, Austrian community pharmacists are currently not authorized to independently discontinue or modify pharmacotherapy. Therefore, the observed reduction likely reflects the successful implementation of pharmacist recommendations by treating physicians. This underlines the potential impact of pharmacist-led interventions, even within regulatory constraints, and highlights the importance of structured collaboration and communication between healthcare professionals (30).

Medication reviews also assessed DRPs, including incorrect therapeutic regimens (i.e., double prescription, dosage errors, inappropriate therapy duration), administration-related problems (i.e., inappropriate pharmaceutical forms, application difficulties), and improper storage of medicines. Although these DRP categories were not formally analyzed by inferential statistics, one may observe a numeric reduction after a medication review. Of note, they are recognized contributors to adverse drug events, especially in vulnerable populations where minor deviations can have serious consequences (31). Dosing errors are a leading cause of medication-related harm, improper storage conditions can impair drug stability and effectiveness, and administration-related problems can introduce non-adherence or result in treatment discontinuation (31, 32). Therefore, detecting such problems may help to identify risk factors and prevent harm in patients.

The conduct of a second medication review did not significantly reduce DRPs compared to a single medication review in the control group. The time span between the visits in this study was relatively short, which may have limited the impact of the second intervention. In addition, the significant improvements already achieved through the first medication review may have resulted in a possible ceiling effect, whereby most optimizations had already been implemented. However, there are currently no data that could inform the optimal frequency of medication reviews. The necessity of a medication review should be assessed on a case-by-case basis. Subsequent medication reviews could be triggered by, e.g., a relevant medical event or a significant change in pharmacotherapy. For instance, Mantzourani et al. reported a significant benefit and a lower re-hospitalization rate if a similar intervention was conducted within 90 days of hospital discharge, which seems plausible, given that pharmacotherapy is frequently adapted in hospital (33). Furthermore, in our study, the first and the second medication review significantly improved health literacy. Thus, patients with poor health literacy at baseline may also benefit from a second medication review.

The limitations of this study include that DRPs are a surrogate parameter. The inclusion of non-selected all-comers and a relatively small sample size within a complex study design precluded the inclusion of well-established laboratory parameters as endpoints (i.e., HbA1c, blood pressure) or even clinical endpoints (e.g., hospitalizations). A possible systematic bias may be that patients in the intervention group consulted their treating physicians more frequently following the intervention and the observed effect sizes might not be attributable to the medication review alone. All study sites were in the city of Vienna and inclusion of study centers in other geographical regions, e.g., rural regions, other countries, would further strengthen the evidence on medication reviews. The number of approached patients was not sufficiently documented. Hence, we cannot make any comments on the response rate of patients to this offer. A cost-utility analysis was not performed. However, cost effectiveness of medication review services has already been demonstrated (22).

## Conclusion

Medication reviews may represent a promising intervention to combat the growing challenge of polypharmacy. Future studies should focus on clinical endpoints and cost-effectiveness of the intervention.

## Data Availability

The original contributions presented in the study are included in the article/Supplementary material, further inquiries can be directed to the corresponding author.

## Footnotes

^1^ www.austria-codex.at
