# Supplementary material for: Effects of a community pharmacy-based structured medication review on drug-related problems in all-comers with polypharmacy: a randomized, controlled, double-blind, parallel-group trial
Source: Front Med (Lausanne). 2025 Sep 3;12:1656595. doi: 10.3389/fmed.2025.1656595 (PMC12440987; doi:10.3389/fmed.2025.1656595)
Supplement: Supplementary file 1 [file Table_1.docx]

**Effects of a community pharmacy-based structured medication review on drug-related problems in all-comers with polypharmacy: A randomised, controlled, double-blind, parallel-group trial**

**Supplementary materials**

# Table S1: Inclusion and exclusion criteria of the study population

**Inclusion criteria**

• Patients with intake of ≥8 systemically available drugs (in case of combination products, ≥8 active ingredients, also including over the counter drugs, especially if they are on the list of interaction relevant over the counter drugs)

• Patients >18 years of age

• Patients signing informed consent

**Exclusion criteria**

• Patients not willing to adhere with the study’s requirements

• Patients unable to understand the nature and purpose of the study

• Previous participation in a structured medication analysis type 2a

# Table S2: Software-supported structured medication review

**Personal questions:**

Name, phone number, address, date of birth, sex, weight, smoking status, allergies.

**General questions:**

1. Would you say that in general your health is?

- Very good
- Good
- Moderate
- Poor
- Very poor

1. Would you say your health recently

- Deteriorated
- Remained unchanged
- Improved

1. How do you rate your medication – Do your drugs work as you expect them to?

- Yes
- Partly DRP: Patient-reported effectiveness
- No DRP: Patient-reported effectiveness

1. How do you tolerate your medications?

- Very good
- Good
- Moderate DRP: Patient-reported tolerability
- Poor DRP: Patient-reported tolerability
- Very poor DRP: Patient-reported tolerability

1. Anyone can forget something every once in a while. How often have you forgotten to take your medicine in the last month?

- Often DRP: Therapy adherence
- Seldom DRP: Therapy adherence
- Never

1. Is there a possibility that you could mistake several drugs?

- Yes DRP: Therapy adherence
- No

1. Under what conditions do you store your medication?

- Proper storage conditions
- Improper storage conditions DRP: Improper storage condition

**Questions for each drug:**

1. What kind of medication is it?

- Long-term medication
- Short-term medication (e.g. of an acute disease)
- On demand medication/emergency medication
  - Inappropriate therapy duration DRP: Inappropriate therapy duration

1. Do you know the indication of the drug?

- Yes, is conclusively known
- No, education required DRP: Health literacy

1. What dosage of this medication do you take? The prescribed dose is available to pharmacists within the electronic software (multiple selections possible)

- Correct
- Unknown to patient DRP: Health literacy
- Deviates from prescription DRP: Dosage error - prescription
- Deviates from SmPC DRP: Dosage error - SmPC

Subcategories: (multiple selections possible)

- - Overdose
  - Underdose
  - Incorrect time of usage
  - Incorrect dosage interval

1. Is there any problem with the use or application of the medication? (multiple selections possible)

- No problems
- Irregular use DRP: Therapy adherence
- Problems with the use of medication DRP: Problems with the use of medication
  - Packaging
  - Handling
  - Application
  - Package leaflet/information text (=SmPC)
- Inappropriate pharmaceutical form DRP: Inappropriate pharmaceutical form
- (lack of) effect DRP: (Lack of) effect
- Side effect/intolerance DRP: Tolerability
- Other DRP: Other problems

1. Are there any contraindications for specific medications?

- No contraindications apply
- Contraindications apply DRP: Contraindicated medication

**Overall medication assessment**

1. Checking double medications DRP: Duplicate prescriptions
2. Checking the individual pDDIs DRP: Clinically relevant pDDIs

List of all potential drug-drug interactions with brief descriptions of the interaction ranked by clinical significance (1: contraindication, 2: severe, 3: moderate, 4: minor interaction*) with checkbox to exclude, if deemed clinically not relevant.

# DRP = drug-related problems, pDDIs = potential drug-drug-interaction, SmPC = summary of product characteristics; *Less severe potential drug-drug-interactions (5-7) were not included in the software

# Table S3: Full list of the primary and all secondary endpoints

**Primary endpoint analysis:**

- The effect of a medication review at baseline (medication review vs. no medication review) on DRPs (at month three-to-four) will be compared by an ANCOVA-like Poisson Regression with treatment group and gender as fixed factors and age in years, baseline number of medications, baseline DRPs as covariates, and pharmacy (in which the patients participated in the study) as random intercept.

**Secondary endpoint analyses:**

- The effect of one medication review at month three-to-four vs. two medication reviews (at baseline and at month three-to-four) on DRPs at month six-to-nine will be compared by an ANCOVA-like Poisson Regression with treatment group and gender as fixed factors and age in years, baseline number of medications, baseline DRPs as covariates, and pharmacy (in which the patients participated in the study) as random intercept.
- Self-reported therapy adherence related DRPs will be analysed by ANCOVA-like Poisson regressions to compare time points Baseline vs. month three-to-four (one medication review vs. no medication review) and baseline vs. month six-to-nine (two medication reviews vs. one medication review), respectively (with treatment group and gender as fixed factors and age in years, baseline number of medications, baseline therapy adherence as covariates, and pharmacy (in which the patients participated in the study) as random intercept.).
- Self-reported health literacy will be analysed by ANCOVA-like Poisson regressions to compare time points Baseline vs. month three-to-four (one medication review vs. no medication review) and baseline vs. month six-to-nine (two medication reviews vs. one medication review), respectively (with treatment group and gender as fixed factors and age in years, baseline number of medications, baseline health literacy as covariates, and pharmacy (in which the patients participated in the study) as random intercept.).
- Relative and absolute frequency of contraindications or severe drug-related problems that require intervention by clinical pharmacologists/clinical pharmacists or treating physicians
- Number of DRPs
- Absolute and relative number of contributors to DRPs, for instance double prescriptions, dosing errors, wrong drug formulation, etc. and the impact of medication reviews on these parameters (analysed descriptively by comparing absolute and relative number of contributors to DRPs between groups)
- Associations of gender, age and number of medications at baseline with the number of DRPs at baseline will be analysed with the Spearman rank correlation coefficient.
- Change in the number of medications from baseline to month three-to-four and from baseline to month six-to-nine will be analysed with an ANCOVA (with treatment group as fixed factor, number of medications at baseline as covariate, and pharmacy (in which the patients participated in the study) as random intercept.).
- Change in DRPs (or contributors) within each group from baseline to month three-to-four or six-to-nine will be analysed using non-parametric, pairwise comparisons.
- Descriptive statistics of patients participating in part 2 of the study per group and presentations of reasons why the study was terminated before part 1.

# ANCOVA = analysis of covariance, DRPs = drug-related problems

# Table S4: Demographics and baseline data for patients who completed Part 1 and 2 of the study

| **Parameter** |  | **Overall** (N = 141) | **Intervention** (N = 74) | **Control** (N = 67) |
| --- | --- | --- | --- | --- |
| Age | Mean ± SD | 68.8 | 67.6 ± 13.7 | 70.2 ± 14.7 |
| Sex (male) | N (%) | 52 (37) | 29 (39) | 23 (34) |
| Weight [kg] | Mean ± SD | 80.8 ± 19.2 | 79.7 ± 19.7 | 82.0 ± 18.8 |
| Smoker | N (%) | 26 (18) | 14 (19) | 12 (18) |
| Pregnant | N (%) | 0 (0) | 0 (0) | 1 (0.02) |
| Medications | Mean ± SD | 12.5 ± 3.9 | 12.7 ± 4.2 | 12.3 ± 3.6 |
| ≥ 10 medications | N (%) | 90 (64) | 48 (65) | 42 (63) |
| ≤ 10 medications | N (%) | 51 (36) | 26 (35) | 25 (37) |
| Active ingredients | Mean ± SD | 14.2 ± 4.4 | 14.5 ± 4.8 | 13.9 ± 3.8 |
| ≥ 10 active ingredients | N (%) | 112 (79) | 61 (82) | 51 (76) |
| ≤ 10 active ingredients | N (%) | 29 (21) | 13 (18) | 16 (24) |
| Baseline DRPs | Mean ± SD | 15.1 ± 9.2 | 15.1 ± 9.7 | 15.1 ± 8.6 |
| Subjective health situation |  |  |  |  |
| Very good | % | 2 | 4 | 0 |
| Good | % | 21 | 20 | 22 |
| Moderate | % | 54 | 50 | 58 |
| Bad | % | 18 | 18 | 18 |
| Very bad | % | 5 | 8 | 2 |
| Subjective health situation changed |  |  |  |  |
| Improved | % | 14 | 15 | 12 |
| Constant | % | 48 | 45 | 51 |
| Deteriorated | % | 39 | 41 | 37 |

# DRP = drug-related problem, SD = standard deviation

# Table S5: Negative binomial mixed effects regression model of the effect of a single medication review compared to no medication review on DRPs (primary endpoint)

| **Independent variables** | **Effect sizes (95% CI)** | **p value** |
| --- | --- | --- |
| Intervention vs. Control group | 0.30 (0.27-0.34) | <0.001 |
| Number of DRPs at baseline | 1.04 (1.03-1.05) | <0.001 |
| Number of active ingredients at baseline | 1.02 (1.00-1.03) | 0.04 |
| Age | 1.00 (1.00-1.01) | 0.37 |
| Sex (male) | 1.09 (0.97-1.23) | 0.17 |

# CI = confidence intervals, DRP = drug-related problem

# Table S6: Negative binomial mixed effects regression model of the effect of a single medication review compared to no medication review on DRPs, excluding the component “clinically relevant pDDIs”

| **Independent variables** | **Effect sizes (95% CI)** | **p value** |
| --- | --- | --- |
| Intervention vs. Control group | 0.34 (0.29-0.39) | <0.001 |
| Number of DRPs at baseline | 1.07 (1.06-1.08) | <0.001 |
| Number of active ingredients at baseline | 1.02 (1.00-1.03) | 0.06 |
| Age | 1.00 (1.00-1.01) | 0.50 |
| Sex (male) | 1.05 (0.92-1.21) | 0.45 |

# CI = confidence intervals, DRP = drug-related problem, pDDI = potential drug-drug-interactions

# Table S7: Negative binomial mixed effects regression model of the effect of two medication reviews compared to one medication review on DRPs

| **Independent variables** | **Effect sizes (95% CI)** | **p value** |
| --- | --- | --- |
| Intervention vs. Control group | 0.80 (0.61-1.04) | 0.10 |
| Number of DRPs at baseline | 1.03 (1.01-1.05) | 0.003 |
| Number of active ingredients at baseline | 1.05 (1.01-1.09) | 0.01 |
| Age | 1.00 (0.99-1.01) | 0.40 |
| Sex (male) | 0.89 (0.67-1.19) | 0.43 |

# CI = confidence intervals, DRP = drug-related problem

# Table S8: Negative binomial mixed effects regression model of the effect of a single medication review compared to no medication review on DRPS related to therapy adherence

| **Independent variables** | **Effect sizes (95% CI)** | **p value** |
| --- | --- | --- |
| Intervention vs. Control group | 0.40 (0.26-0.61) | <0.001 |
| Therapy adherence at baseline | 1.75 (1.50-2.05) | <0.001 |
| Number of active ingredients at baseline | 0.99 (0.95-1.04) | 0.73 |
| Age | 1.01 (1.00 -1.03) | 0.16 |
| Sex (male) | 0.72 (0.47-1.10) | 0.13 |

# CI = confidence intervals, DRP = drug-related problem

# Table S9: Negative binomial mixed effects regression model of the effect of two medication reviews compared to one medication review on DRPs related to therapy adherence

| **Independent variables** | **Effect sizes (95% CI)** | **p value** |
| --- | --- | --- |
| Intervention vs. Control group | 1.51 (0.76-2.98) | 0.24 |
| Therapy adherence at baseline | 1.52 (1.12-2.07) | 0.007 |
| Number of active ingredients at baseline | 1.00 (0.93-1.07) | 0.97 |
| Age | 1.00 (0.98-1.02) | 0.95 |
| Sex (male) | 1.09 (0.56-2.12) | 0.80 |

# CI = confidence intervals, DRP = drug-related problem

# Table S10: Negative binomial mixed effects regression model of the effect of a single medication review compared to no medication review on DRPs related to health literacy

| **Independent variables** | **Effect sizes (95% CI)** | **p value** |
| --- | --- | --- |
| Intervention vs. Control group | 0.36 (0.22-0.59) | <0.001 |
| Health literacy at baseline | 1.37 (1.25-1.50) | <0.001 |
| Number of active ingredients at baseline | 1.02 (0.96-1.08) | 0.54 |
| Age | 1.02 (1.00-1.04) | 0.06 |
| Sex (male) | 1.17 (0.72-1.90) | 0.51 |

# CI = confidence intervals, DRP = drug-related problem

# Table S11: Negative binomial mixed effects regression model of the effect of two medication reviews compared to one medication review on DRPs related to health literacy

| **Independent variables** | **Effect sizes (95% CI)** | **p value** |
| --- | --- | --- |
| Intervention vs. Control group | 0.31 (0.10-0.93) | 0.04 |
| Health literacy at baseline | 1.30 (1.07-1.58) | 0.01 |
| Number of active ingredients at baseline | 0.91 (0.78-1.07) | 0.26 |
| Age | 1.00 (0.95-1.05) | 0.98 |
| Sex (male) | 0.90 (0.29-2.81) | 0.86 |

# CI = confidence intervals, DRP = drug-related problem

# Table S12: Negative binomial mixed effects regression model of the effect of a single medication review compared to no medication review on the number of medications

| **Independent variables** | **Effect sizes (95% CI)** | **p value** |
| --- | --- | --- |
| Intervention vs. Control group | 0.90 (0.83-0.98) | 0.012 |
| Number of medications at baseline | 1.06 (1.05-1.07) | <0.001 |
| Age | 1.00 (1.00-1.01) | 0.23 |
| Sex (male) | 1.01 (0.93-1.10) | 0.74 |

# CI = confidence intervals

# Table S13: Negative binomial mixed effects regression model of the effect of two medication reviews compared to one medication review on the number of medications

| **Independent variables** | **Effect sizes (95% CI)** | **p value** |
| --- | --- | --- |
| Intervention vs. Control group | 0.96 (0.87-1.07) | 0.48 |
| Number of medications at baseline | 1.07 (1.05-1.08) | <0.001 |
| Age | 1.01 (1.00-1.01) | 0.01 |
| Sex (male) | 1.01 (0.91-1.13) | 0.83 |

# CI = confidence intervals

# Table S14: Negative binomial mixed effects regression model of the effect of a single medication review compared to no medication review on the number of active ingredients

| **Independent variables** | **Effect sizes (95% CI)** | **p value** |
| --- | --- | --- |
| Intervention vs. Control group | 0.91 (0.84-0.98) | 0.01 |
| Number of active ingredients at baseline | 1.05 (1.04-1.06) | <0.001 |
| Age | 1.00 (1.00-1.00) | 0.20 |
| Sex (male) | 1.01 (0.93-1.09) | 0.79 |

# CI = confidence intervals

# Table S15: Negative binomial mixed effects regression model of the effect of two medication reviews compared to one medication review on the number of active ingredients

| **Independent variables** | **Effect sizes (95% CI)** | **p value** |
| --- | --- | --- |
| Intervention vs. Control group | 0.96 (0.87-1.06) | 0.42 |
| Number of active ingredients at baseline | 1.06 (1.05-1.07) | <0.001 |
| Age | 1.00 (1.00-1.01) | 0.02 |
| Sex (male) | 1.00 (0.91-1.11) | 0.94 |

# CI = confidence intervals

# Table S16: Logistic mixed effects regression model of the effect of a single medication review compared to no medication review on patient-reported health, “very good” or “good” vs. “moderate” or “bad” or “very bad” (post-hoc)

| **Independent variables** | **Effect sizes (95% CI)** | **p value** |
| --- | --- | --- |
| Intervention vs. Control group | 2.20 (1.08-4.48) | 0.03 |
| Patient-reported health at baseline | 11.29 (5.14-24.80) | <0.001 |
| Number of active ingredients at baseline | 0.860 (0.78-0.95) | 0.003 |
| Age | 0.987 (0.96-1.01) | 0.31 |
| Sex (male) | 1.32 (0.65-2.70) | 0.45 |

# CI = confidence intervals

# Table S17: Logistic mixed effects regression model of the effect of a single medication review compared to no medication review on patient-reported change in health, “improved” vs. “constant” or “detoriated” (post-hoc)

| **Independent variables** | **Effect sizes (95% CI)** | **p value** |
| --- | --- | --- |
| Intervention vs. Control group | 3.09 (1.40-6.81) | 0.005 |
| Patient-reported change in health at baseline | 3.84 (1.40-10.57) | 0.009 |
| Number of active ingredients at baseline | 1.02 (0.94-1.11) | 0.58 |
| Age | 0.98 (0.96-1.01) | 0.26 |
| Sex (male) | 0.87 (0.40-1.87) | 0.72 |

# CI = confidence intervals
